# Soil nutrient adequacy for optimal cassava growth, implications on cyanogenic glucoside production: A case of konzo-affected Mtwara region, Tanzania

**DOI:** 10.1371/journal.pone.0216708

**Published:** 2019-05-13

**Authors:** Matema L. E. Imakumbili, Ernest Semu, Johnson M. R. Semoka, Adebayo Abass, Geoffrey Mkamilo

**Affiliations:** 1 Department of Soils and Geological Sciences, Sokoine University of Agriculture, Morogoro, Tanzania; 2 The International Institute of Tropical Agriculture, Dar es Salaam, Tanzania; 3 Roots and Tubers Department, Naliendele Agricultural Research Institute, Mtwara, Tanzania; Ohio State University South Centers, UNITED STATES

## Abstract

Soils in areas affected by konzo (a cassava cyanide intoxication paralytic disorder) are predominantly infertile and probably unable to supply cultivated cassava with the nutrients it needs to achieve optimal growth. Soil nutrient supply in these areas could also be influencing cyanogenic glucoside production in cassava, however there is hardly any knowledge on this. An assessment of soil nutrient levels on crop fields in konzo-affected areas was therefore carried out to determine their adequacy for optimal cassava growth. Konzo-affected Mtwara region of Tanzania, was used as a case study. Whether soil nutrient supply influences cyanogenic glucoside production in cassava cultivated in konzo-affected areas and how it could be doing this, was additionally investigated. To investigate this, correlations between total hydrogen cyanide (HCN) levels (a measure of cyanogenic glucoside content) in cassava roots and various soil nutrient levels on crops fields were carried out. This was followed by an investigation of relationships between cases of cassava cyanide intoxication and soil nutrient levels on crop fields from which the consumed toxic cassava roots had been harvested. Cases of cassava cyanide intoxication were used as a proxy for high cyanogenic glucoside levels in cassava roots. Logistic regression analysis was used in the latter investigation. Other important non-nutrient soil chemical characteristics, like pH and soil organic carbon, were also included in all analysis performed. The results revealed that most soil nutrients known to have reducing effects on cassava cyanogenic glucosides, like potassium (mean = 0.09 cmol/kg, SD = 0.05 cmol/kg), magnesium (mean = 0.26 cmol/kg, SD = 0.14 cmol/kg) and zinc (mean = 1.34 mg/kg, SD = 0.26 mg/kg) were deficient on several crop fields. The results also showed that cyanogenic glucosides in cassava roots could be increased with the increased supply of sulphur in soils in bitter cassava varieties (r_s_ = 0.593, p = 0.032), and with the increased supply of P in soils in all cassava varieties (r_s_ = 0.486, p = 0.026). The risk of cassava cyanide intoxication occurring (and thus high cyanogenic glucoside levels in cassava) was found to be likely increased by cultivating cassava on soils with high pH (X^2^ = 8.124, p = 0.004) and high iron (X^2^ = 5.740, p = 0.017). The study managed to establish that cassava grows under conditions of severe nutrient stress and that soil nutrient supply influences cyanogenic glucoside production in cassava cultivated in konzo-affected areas of Mtwara region. Despite the multiple soil nutrient deficiencies on crop fields, low soil fertility was however not the only probable cause of increased cyanogenic glucosides in cassava, as high soil nutrient levels were also found to be potential contributors.

## Introduction

Crop yields are greatly reduced by low soil fertility. The reduction in yields is caused by an inadequate supply of much needed nutrients to growing plants. Besides limiting yields, low soil fertility further affects the nutritional composition of crops, altering their nutritional quality. Yields of hardy crops like cassava (*Manihot esculenta* Crantz) are also reduced by low soil fertility. The cyanogenic glucoside content of cassava is also probably negatively affected by low soil fertility. Cyanogenic glucosides occur naturally in cassava and are an important nutritional quality determining factor in its edible parts. Levels of cyanogenic glucosides need to be very low in fresh cassava roots or in cassava products, if these foods are to be considered innocuous and safe for consumption. Ingestion of high amounts of cyanogenic glucosides exposes humans to cyanide intoxication, which can have detrimental effects on their health. Although still uncertain of this, the Codex Alimentarius Commission currently recommends total hydrogen cyanide (HCN) levels (a measure of cyanogenic glucoside content) of less than 50 mg/kg in fresh cassava roots, as safe for consumption [1].

Due to its cyanogenic glucosides, cassava has many at times resulted in cases of cyanide intoxication in a number of cassava dependent, rural poor communities in sub-Saharan African. During periods of high dependence on cassava, ingestion of cyanides in these communities is estimated to be as high as 15 to 50 mg/day [2]. These levels are quite high, particularly for children because of their lower body weights and higher nutritional needs. According to the current health guidelines, in order to avoid acute intoxication, cyanide ingestion should not exceed 0.09 mg/kg of body weight within a short-time interval and to avoid chronic intoxication, cyanide ingestion should not exceed 0.02 mg/kg of body weight each day [3]. Cases of cassava cyanide intoxication in cassava dependent communities, have often resulted in a health disorder called konzo (spastic paraparesis), which causes an irreversible paralysis of legs in affected individuals [4,5]. A number of sub-Saharan African countries have been affected by konzo, and the disorder is reported as persistent in very deprived areas of Mozambique, the Democratic Republic of Congo (DRC), Tanzania [6], Central African Republic [7,8] and in eastern Cameroon [9,10].

A characteristic common to all areas affected by konzo is their low soil fertility. Except for konzo-affected areas in DRC and Cameroon, all other konzo-affected areas are situated along the coast or along a lake shore. They include; the coastal districts of Nampula and Zambézia provinces in Mozambique [11], Newala and Mtwara districts in Tanzania [12] and lake shore Tarime district in Tanzania [13]. All mentioned areas are dominated by sandy soils with very low soil fertility [13–15]. Although not coastal, konzo-affected areas in Bandundu province in DRC, located in the Savannah zone, primarily consist of relatively infertile soils [16]. Soils in the non-coastal Eastern region of Cameroon are additionally described as having sandy clay textures and as being largely degraded [17]. Soils in konzo-affected western Central African Republic, bordering the eastern region of Cameroon [9], are probably just as degraded and unsuitable for crop production.

**Fig 1:**
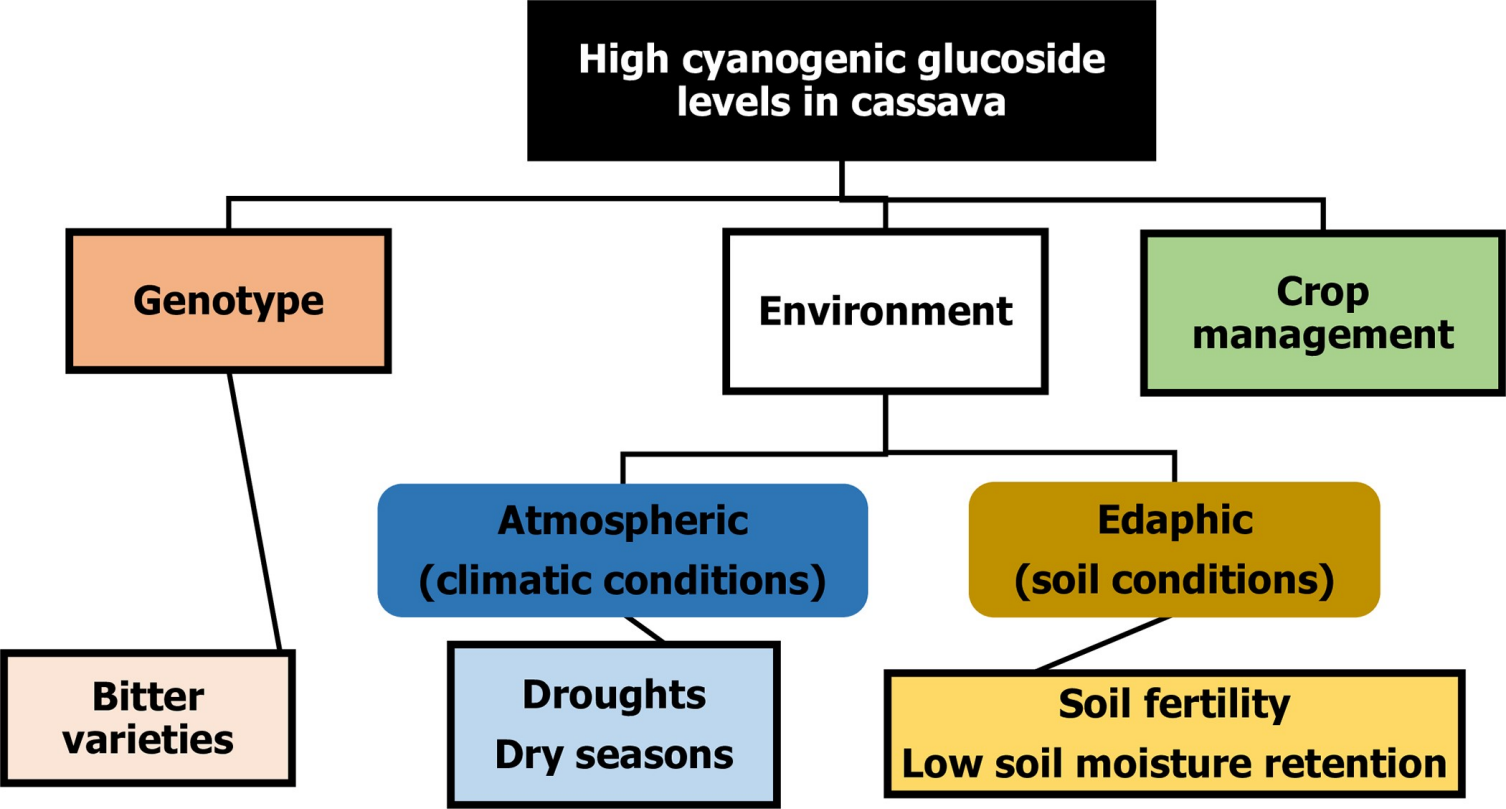
Commonly known and less known agronomic reasons for high cyanogenic glucoside levels in cassava.

The agronomic factors that lead to increased cyanogenic glucoside levels and exacerbate cyanide intoxication during konzo outbreaks include; farming systems dominated by highly toxic bitter cassava varieties and drought or dry season related water stress [11,12,18] (Fig 1). While the sandy nature of soils in most konzo-affected areas contributes to water stress, the additional influence of soil nutrient supply on cassava cyanogenic glucoside production, from these predominantly nutrient poor soils, cannot be ignored and needs to be investigated. A study was hence carried out to assess the adequacy of soil nutrients for optimal cassava growth in konzo-affected areas. The study additionally investigated whether soil nutrient supply influences cyanogenic glucoside production in cassava cultivated in these areas and how it could be doing this. Konzo-affected Mtwara region of Tanzania, was used as a case study.

In order to investigate whether soil nutrient supply influences cassava cyanogenic glucoside production in konzo-affected areas and how it could be doing this, relationships between soil nutrient levels on crop fields and total root HCN levels in cassava cultivated on them, were investigated. The hypothesis tested was that *‘there is no association between levels of various soil nutrients on crop fields and root HCN levels of cassava cultivated on them’*. This was followed by an investigation of the soil nutrients likely responsible for increasing the risk of cassava cyanide intoxication. Relationships between cases of cassava cyanide intoxication and soil nutrient levels on crop fields from which the consumed toxic roots had been harvested, were analysed. Cassava cyanide intoxication was used as a proxy for high levels of cyanogenic glucosides in cassava roots in this analysis. The hypothesis tested in the latter investigation was that *‘the likelihood of the occurrence of cassava cyanide intoxication is related to soil nutrient levels on crop fields on which cassava is cultivated’*. Information on cassava cyanide intoxication experiences had to be collected, in order to conduct this latter investigation. However, because most cassava cyanide intoxication experiences may have occurred earlier and not in the year that the present study was conducted, an assumption had to be made with regard to the assessed soil nutrient levels on crop fields. The study had to assume that soil nutrients on crop fields had remained relatively the same from the time of each intoxication experience. Relationships drawn-out between soil nutrient levels and cassava cyanide intoxication, were hence assumed to be comparable to those present at the time of the intoxication experiences.

## Materials and methods

### Description of study area

The study was carried out in areas previously reported to have been affected by konzo in Tanzania’s Mtwara region (S 10^o^16'25"; E 40^o^10'58"). The districts Masasi, Mtwara rural and Newala are the three districts reported to have been affected by konzo in Mtwara region [12]. The study however focused on the districts Mtwara rural and Newala, which are only two out of the five districts of Mtwara region. Mtwara rural district and Newala district both lie in Tanzania’s, Coastal lowlands agroecological zone (C2). Both districts lie on the Makonde plateau. The region is characterised by mono-modal rainfall, which ranges from 800 to 1000 mm/year and by maximum and minimum temperatures that vary between 29 and 31 ^o^C and between 19 and 23 ^o^C, respectively [19]. Soils in Mtwara region are generally classified as Ferralic Cambisols and are predominantly sandy [19].

### Selection of survey participants

The survey was carried out from the 7^th^ to the 16^th^ of October, 2014, during the hot-dry season, which is a common harvest time for cassava in Mtwara region. Eight villages had been included in the survey. The villages had been selected from the 18 villages visited during a konzo rehabilitation and prevention program, carried out from 2008 to 2009 by the Tanzania Food and Nutrition Centre (TFNC) and the Tanzania Red Cross Society (TRCS), with technical support from the Australian National University (ANU) [12]. Four villages were selected from Mtwara rural district and another four from Newala district using simple random sampling [20]. To do this, villages from each district were numbered and randomised using the ‘RANDBETWEEN’ function in Microsoft Excel [21]. The first four villages that appeared for each district in the randomised list were then picked. Mdimba, Ngalu, Songambele and Mkunjo were the villages selected from Newala district, whereas the villages Njengwa, Nyundo, Niyumba and Kiromba were selected from Mtwara rural district. Using simple random sampling, 15 households were then randomly picked from each selected village to participate in the survey. A 2012 village census list was used to select the households. The census list was the most recent of the past census carried out nationwide in Tanzania. Each household in a village listed in the census list was given a unique number to identify it. The numbered list was then randomised using the ‘RANDBETWEEN’ function in Microsoft Excel. The first 15 households on the randomised list were then selected. A total of 120 households were selected, 61 from the konzo-affected villages of Newala district and 59 from the konzo-affected villages of Mtwara rural district.

Permission to conduct the study was sought from the regional government administrative offices of Mtwara region, prior to the survey. Permission had been granted and put in writing. They had made no request for additional permissions for ethical clearance from the Tanzania Commission for Science and Technology (COSTECH). COSTECH is the institution that acts as a research review board in Tanzania. To the regional offices understanding, the research did not threaten to breach any ethics. The letter collected from the regional office was presented to the district administrative offices in Newala and Mtwara rural districts. After obtaining verbal acknowledgment and consent to proceed with the research from the two district administrative offices, the letter obtained from the regional office was then presented to each village administrative committee from the villages included in the study. The research was explained to the village administrative committees and later to the households selected to participate in the study. They all verbally consented for the work to proceed and to take part in the study.

A letter of introduction had also been initially obtained from Sokoine University of Agriculture, which was the host institution for the principal researcher. The letter stated the principal researchers’ name, the institution they belonged to and the nature of the research to be carried-out. An additional humble request for the researcher to be kindly supported, had also been included.

### Farmer interviews

Household heads (farmers) were individually interviewed to obtain information on the households’ cassava cyanide intoxication experiences (see S1 Text). If a household had both spouses present, either spouse was interviewed. The farmers were asked whether any member of their household had experienced anything strange or any difficulties after consuming cassava. The mention of any of the following symptoms was checked; dizziness, headaches, stomach pains, nausea, vomiting, brief confusion, difficulty in standing, difficulty in speaking (a heavy tongue) or even death [2,22]. Clinical validation of symptoms experienced had not been obtained, the responses were hence based on the farmers own understanding of cassava cyanide intoxication. All farmers however appeared to be very aware of cassava cyanide intoxication and its associated symptoms. The education given on cassava cyanide toxicity during the konzo prevention and rehabilitation programs probably helped increase the people’s awareness on the issue. Farmers that agreed to have had at least one household member affected by cyanide intoxication after consuming cassava, were also asked whether the cassava roots that had brought about the intoxication had been obtained from the households’ own crop field. In order to link the cyanide intoxication cases to soil nutrient levels on crop fields from which the poisonous cassava roots had been harvested, farmers were also asked whether they still cultivated the crop field from which the toxic cassava roots had been harvested. Soil samples were then collected from the fields cropped at the time of the intoxication experience. Soils from crop fields of households not affected by cassava cyanide intoxication in the konzo-affected villages of Mtwara rural and Newala districts were also collected.

Apart from the information on cassava cyanide intoxication, information on soil nutrient management practices used by farmers to maintain their crop fields was additionally collected. The information sheds light on the fertility status of soils on crop fields on which cassava is cultivated, in konzo-affected areas of Mtwara region. The information is shown in Table 1.

**Table 1:** Soil nutrient management practices used on cassava crop fields.

Soil nutrient management practices	Mtwara rural district	Newala district	Both districts
	n = 59	n = 61	n = 120
1. Farmers using the slash and burn practice	98.3%	98.4%	98.3%
2. Length of crop field cultivation periods:			
average period used	4 years	3 years	4 years
shortest period used	2 years	2 years	2 years
longest period used	6 years	6 years	6 years
3. Length of fallow periods:			
average period used	3 years	2 years	2 years
shortest period used	1 year	1 year	1 year
longest period used	8 years	6 years	8 years
4. Farmers practicing:			
cassava mono-cropping	8.6%	3.3%	5.9%
cassava mixed-cropping	91.4%	96.7%	94.1%
5. Farmers using:			
inorganic fertilisers	1.7%	21.7%	11.8%
animal manure	1.7%	11.5%	6.7%
6. Farmers intercropping cassava with cashew	77.4%	42.4%	58.9%
7. Farmers sulphur dusting their cashew	36.4%	75.0%	51.4%

Values representing number of years have been rounded off for a realistic representation of the time periods.

All values in percentages were calculated out of n = 59, 61 and 120 for values recorded for Mtwara rural district, Newala district and for both districts combined, respectively.

The farmers mainly practiced slash and burn semi-permanent cultivation, which is a downgraded form of the slash and burn shifting cultivation practice, with fallow periods that are less than double the length crop fields are continuously cultivated. The farmers described the cultivation practice as their ‘fertiliser’. The short fallow periods used however do not allow forest recovery and adequate soil fertility restoration. Due to the limited supply of land, some farmers could not shift from one field to the next and were thus left to continuously crop the same piece of land year after year. These farmers however normally divided their one piece of land into at least three to four portions, which could alternately be cultivated and fallowed. Using their land in this way enabled them to cultivate the same crop field year after year, while still giving the land a chance to get restored to a certain degree. The length of time that crop fields could be continuously cultivated in these areas was however getting shorter, to as low as 2 years (Table 1). Most farmers complained that their crop fields could not be continuously cultivated for long, as the soils easily got ‘fatigued’ (lost their productivity).

The use of either organic or inorganic fertilisers was low in these areas and fertiliser use was generally better in Newala district compared to Mtwara rural district (Table 1). Urea and ammonium sulphate were the two main inorganic fertilisers used, while goat manure was the only organic fertiliser used by the farmers. The use of ammonium sulphate was however being discouraged as it had been observed to be causing soil infertility. The fertilisers were however not applied on cassava but on maize (*Zea mays*). Cassava only got to indirectly benefit from fertiliser application either as an intercrop with maize or as a crop planted in sequence with maize. Due to land shortages some farmers cropped cassava with cashew trees (*Anacardium occidentale*), this was however more common when the cashew trees were still young. A good number of farmers dusted their cashew trees with sulphur (Table 1). Cassava cropped with cashew trees hence got an additional supply of S from the S that fell onto the soil surface during sulphur dusting. Sulphur was mainly being used to control powdery mildew on cashew trees.

Almost all farmers practiced mixed cropping, cassava was hence mostly grown with other crops on crop fields (Table 1). Mixed cropping is a common practice in traditional African cropping systems. Maize, sorghum (*Sorghum bicolor*), rice (*Oryza sativa*) and cowpeas (*Vigna unguiculata*) were the crops commonly cropped together with cassava on crop fields from Mtwara rural. In Newala district, maize, sorghum, millet and pigeon peas (*Cajanus cajan*) were most commonly cropped with cassava. No clarification was made on the type of millets grown, it is thus unclear whether it was finger millet (*Eleusine coracana*) or the bulrush millets (for example, *Pennisetum glaucum*) that were cultivated. As for weeding, about half of the farmers interviewed kept cassava well weeded by weeding it twice or thrice in the first three months after it had been planted.

### Soil sample collection, preparation and analyses

In order to assess soil nutrient levels in konzo-affected areas of Mtwara region, composite soil samples of about 500 g were collected from the farmers’ crop fields [dx.doi.org/10.17504/protocols.io.x72frqe]. This was done by collecting soil from the top 20 cm layer of the soil surface, from at least 10 randomly selected points on each crop field [23,24]. Soil samples had however been collected from only 112 crop fields out of the 120 fields belonging to all the households that participated in the study; 57 and 55 of the soil samples had been taken from Mtwara rural district and Newala district, respectively. To investigate soil nutrient adequacy for optimal cassava growth, the collected soil samples had to be analysed for their soil chemical characteristics. The soil samples were air-dried and later analysed for; soil pH, organic carbon (OC), total nitrogen (N), available phosphorous (P), exchangeable potassium (K), calcium (Ca) and magnesium (Mg), available sulphur (S), available zinc (Zn), copper (Cu), iron (Fe) and manganese (Mn) and exchangeable aluminium (Al).

All laboratory methods used to analyse the various soil chemical characteristics were as follows [25]; Soil pH was determined in a 1:1 soil to water solution and the Bray No. 1 method was used to extract P. Total N was determined using the micro Kjeldhal method and OC using the Walkley and Black dichromate method. The exchangeable cations K, Ca and Mg were extracted using 1 N ammonium acetate (NH_4_OAc) leaching solution, buffered at pH 7. Monocalcium phosphate [Ca(H_2_PO_4_)_2_] extracting solution was used to extract S, while Zn, Cu, Fe and Mn were extracted using diethylenetriaminepentaacetic acid (DTPA). Exchangeable Al was extracted using 1 N potassium chloride (KCl) extracting solution and Al saturation was calculated using Eq 1 [26]. The soil texture of the sampled soils had also been determined and the hydrometer method had been used for this purpose.

$$Al\;saturation\;\left(\%\right)=\left(\frac{Al}{Al+Ca+Mg+K}\right)\;cmol/kg\times 100$$ (1)

### Cassava root sampling and total hydrogen cyanide determination in fresh cassava roots

In order to investigate relationships between soil nutrient levels and the cyanogenic glucoside content of cassava produced in konzo-affected areas, root samples were also collected from crop fields from which the soil samples had been taken. Cassava root samples had however been collected from only 21 fields out of the 112 crop fields from which soil samples had been collected. The picrate papers that were to be used for root HCN determination had gotten spoiled as they were kept in a freezer and not in a deep freezer. This was only discovered right before the survey, hence the failure to collect cassava root samples from each field from which a soil sample had been taken. From the 21 cassava root samples collected, 11 and 10 root samples had been taken from Mtwara rural district and Newala district, respectively. Roots of both commonly cultivated bitter and sweet cassava varieties were sampled to better represent the cyanogenic glucoside content of all cassava roots during a dry season period in konzo-affected areas of Mtwara region. The classification of selected cassava varieties as being bitter or sweet was based on the farmers own traditional classification system, which uses cassava root taste as a class determining factor.

Cassava root samples were collected from four randomly selected plants of the same variety from a farmers’ crop field [dx.doi.org/10.17504/protocols.io.ydxfs7n]. Only plants identified by famers as being ready for harvest and thus ready for consumption were selected. Three roots were sampled per plant for HCN analysis [27]. Note that the cassava roots were harvested from plants of slightly different ages across all sampled crop fields and that crop management practices could have differed from one crop field to the next. Sampled cassava plants were thus exposed to varied growing conditions and could thus have had their cyanogenic glucoside production differently influenced. These differences do not however interfere with the study’s objectives, as the study aims to reflect representative HCN levels in cassava roots consumed during a dry season period in areas affected by konzo.

Total HCN levels of fresh cassava roots were determined using the picrate paper method, as previously mentioned [28,29] [dx.doi.org/10.17504/protocols.io.ygzftx6]. A 100 mg section of fresh cassava root taken from the middle of the root was placed in a vial with buffer solution and a picrate paper. The contents in the vial were then left to incubate in the dark at room temperature for 16 to 24 hours. The picrate papers darkened by the cyanide liberated from the fresh root sections, were then eluted in 5 ml of distilled water. The absorbance of the solution so obtained was then determined using a spectrophotometer at 510 nm. Cassava root HCN content on a fresh weight basis was then calculated using Eq 2. The value of 396 in Eq 2 is a value derived from the calibration curve between picrate solutions placed in known standard cyanide solutions and their absorbance values.

TotalHCN(mg/kg)freshweight=396×absorbance (2)

### Data analysis

Soil nutrient levels on crop fields were analysed using descriptive statistics (means, standard deviations, and minimum and maximum values) [20]. Their adequacy for cassava production was assessed by comparing them to known critical levels and sufficiency ranges recommended for optimal cassava growth. As the data was not normally distributed for cassava root HCN levels, due to the few samples, the Spearman’s correlation (two-tailed) [20] was used to assess the statistical significance of relationships between cassava root HCN levels and various soil nutrients. The risk of cassava cyanide intoxication occurring because of soil nutrient levels on crop fields from which consumed cassava roots are harvested, was examined using the binomial logistic regression analysis [30]. Soil nutrient levels on crop fields of households that had experienced cassava cyanide intoxication and those on fields of households that had not experienced cassava cyanide intoxication were compared in the logistic regression analysis. Important non-nutrient soil chemical characteristics like pH, OC and Al saturation were also included in all analysis conducted. All statistical analyses were carried out using IBM SPSS Statistics, version 20.

## Results and discussion

### Suitability of soils for optimal cassava growth

Table 2 shows the soil nutrient levels on crop fields in konzo-affected areas of Mtwara region, together with their suitability for optimal cassava growth. Levels of other important soil chemical characteristics like pH, OC and Al saturation, are also included.

**Table 2:** Soil chemical characteristics of crop fields located in konzo-affected villages of Mtwara region and their suitability for cassava production.

Soil parameter	Mean	Standard deviation	Minimum		Maximum		Range/level rated as suitable for cassava production	Rated according to:
	n = 112	SD	Value	Rating^‡^	Value	Rating^‡^		
pH	5.29	0.56	4.40	l	6.96	m	4.5–7.0	[26]
OC (%)	0.63	0.27	0.31	vl	1.22	vl	4.0–10.0	[31]
N (%)	0.07	0.03	0.01	vl	0.33	m	0.20–0.50	[31]
P (mg/kg)	8.21	7.62	1.19	l	34.52	h	< 4.2	[32]
K (cmol/kg)	0.09	0.05	0.02	vl	0.32	h	0.15–0.25	[26]
Ca (cmol/kg)	2.02	0.93	0.46	l	4.49	m	1.0–5.0	[26]
Mg (cmol/kg)	0.26	0.14	0.02	vl	0.76	m	0.40–1.00	[26]
S (mg/kg)	5.30	3.17	0.60	l	23.51	h	< 6.0	[31]
Zn (mg/kg)^†^	0.34	0.26	trace	vl	1.79	m	1.0–3.0	[33]
Cu (mg/kg)^†^	0.01	0.05	trace	vl	0.43	m	0.3–0.8	[33]
Fe (mg/kg)^†^	32.87	19.10	3.19	l	83.45	vh	4.0–6.0	[33]
Mn (mg/kg)^†^	9.74	10.03	1.29	m	71.71	vh	1.2–3.5	[33]
Al Saturation (%)^ǂ^	9.27	9.19	0.00	m	32.94	m	< 75.0	[26]

^‡^ vl, l, m, h and vh stand for very low, low, medium, high and very high, respectively.

^ǂ^ For Al saturation, n = 72

^†^ For Zn, Cu, Fe and Mn, n = 111.

The soil texture classes for the sampled soils were as follows; 58.0% Loamy sand, 22.3% Sandy clay loam, 13.4% Sandy loam and 6.3% Sand.

Note that pH 5.0 in 0.01 M CaCl_2_ (1:2) is about pH 5.5 in H_2_O (1:1).

Soil pH levels in the region were mainly in the desirable range and were hence not too low or too high for optimal cassava growth (Table 2). Only 0.9% of the fields had soil pH levels below 4.5, while none had pH levels above 7.0. The pH of soils in Mtwara region is not as low as might be expected for highly weathered soils and is usually more than 5.0 because of the presence of limestone in the parent material of these soils [34]. Limestone has increasing effects on soil pH. All fields had low levels of Al saturation, meaning that aluminium toxicity posed no limitations on the growth of cassava.

Almost all (99.1%) of the fields sampled had soil N levels below what is considered as sufficient for a broad range of tropical crops [31] (Table 2). The low OC levels in all (100%) crop fields together with the low use of fertilisers, explains why N was low in these soils. Soil OC is the main source of N for crops grown without fertiliser application. With regard to cyanogenic glucoside production, some studies have reported that an improved supply of N, on N deficient soils, is able to reduce cassava root HCN levels [35,36]. Other studies have similarly reported reductions in cyanogenic glucosides with improved N supply [37,38]. Improving the supply of N on these N deficient soils could thus be beneficial for reducing cassava root HCN levels. A moderate supply of N would however be better, as a high supply of N could increase cyanogenic glucoside levels [38].

Only 34.8% of the sampled crop fields had soil P levels below the critical level of 4.2 mg/kg (Table 2). Low soil P was hence not so much of a problem on most fields. Not much research has been carried out to determine the relationship between soil P supply and root cyanogenic glucoside production. One study however showed that changes in the supply of P may have no influence on cyanogenic glucoside production in cassava [38]. However, when P is adequate in soils, plant root development is improved, enabling greater water uptake particularly under low soil moisture conditions. Adequate levels of P in soils would thus be beneficial for reducing cyanogenic glucosides in cassava, in areas prone to konzo. Moving to soil K, about 84.3% of the sampled fields had K levels below the range considered adequate for healthy cassava growth. Hence unlike soil P, most crop fields were deficient in K. A few fields however had high levels of K. An adequate supply of K, through fertiliser application has been shown to often reduce HCN levels in cassava roots [39,40]. Low K in soils of konzo-affected Mtwara region could thus contribute to increased cyanogenic glucoside production in cassava. Some studies have however shown no effects on cyanogenic glucosides with an improved supply of K [41]. Reductions in root HCN with improved soil K may thus not always occur.

With only 13.9% of the fields deficient in soil Ca, this nutrient was adequate for cassava production on most crop fields. Adequate levels of Ca in these soils can be attributed to the calcium rich limestone parent material that these soils have been formed from. Reduced root HCN levels are expected in cassava with an adequate supply of Ca [35,42]. In contrast to Ca, the nutrients Mg and Zn were severely deficient in 84.3% and 93.0% of the sampled crop fields, respectively. Like soil Ca, the adequate supply of Mg and Zn in soils, is beneficial for reducing cyanogenic glucosides in cassava [35,43]. This is observed in the reduction of cassava root HCN levels, with the application of ash, which is rich in K, Ca and Mg [43]. Deficiencies of K, Ca, Mg and Zn in soils of konzo-affected areas of Mtwara region could thus result in increased cyanogenic glucoside production in cassava cultivated in these areas.

Sulphur was deficient in 63.5% of the sampled fields, its deficiency on crop fields was thus not as widespread as some other soil nutrients. The practice of sulphur dusting cashew trees could be contributing to improving soil S levels on these crop fields. Reduced root HCN levels with an improved supply of S in soils has been reported in some studies [35,42,44]. Low S levels could thus have an increasing effect on root cyanogenic glucoside production on crop fields. Unlike S, Cu was deficient on almost all (95.7%) of the sampled crop fields (Table 2). On the other hand, none of the fields had low Mn levels and only 0.9% of the fields had low soil Fe levels. Low Fe and Mn were hence not a problem on these soils, but their toxic levels on 89.9% and 52.9% of the crop fields, respectively, could be problematic to the growth of cassava [45] and probably to cyanogenic glucoside production. High levels of Fe are expected, as soils in Mtwara region have ferralic properties and are thus rich in Fe. There are hardly any reports on the influence of supply of Cu, Fe or Mn on cassava root HCN levels. The very low soil Cu levels could however cause nutrient stress in cassava just like the very high levels Fe and Mn.

### Relationships between soil nutrient levels and cassava root cyanogenic glucoside contents

The results of the Spearman’s correlation analyses, carried out to determine relationships between soil nutrients and HCN levels of fresh cassava roots in areas affected by konzo are shown in Table 3. Correlations between soil nutrients and root HCN levels of bitter and sweet cassava varieties are also shown in Table 3.

**Table 3:** Correlations between various soil chemical characteristics on crop fields and root HCN levels in cassava cultivated on them.

Soil parameter	Bitter varieties		Sweet varieties		All varieties	
	(n = 13)		(n = 8)		(n = 21)	
	r_s_	p-value	r_s_	p-value	r_s_	p-value
pH	0.135	0.660	-0.407	0.317	0.072	0.758
OC	0.482	0.095	0.675	0.066	0.149	0.519
N	-0.208	0.496	0.182	0.666	-0.023	0.922
P	0.500	0.082	0.238	0.570	**0.486**	0.026
K	-0.394	0.183	-0.443	0.272	-0.106	0.649
S	**0.595**	0.032	0.714	0.047	0.374	0.095
Ca	0.014	0.964	-0.072	0.865	0.100	0.666
Mg	-0.339	0.257	0.132	0.756	0.018	0.940
Zn	0.262	0.387	0.491	0.217	0.356	0.113
Cu	.	.	-0.082	0.846	-0.222	0.334
Fe	-0.157	0.609	0.333	0.420	-0.014	0.951
Mn	-0.225	0.459	-0.476	0.233	-0.001	0.996
Al Saturation	-0.152	0.774	-0.100	0.873	-0.116	0.733

Values of correlation coefficients (r_s_) in bold are significant at p < 0.05 using the Spearman’s correlation (two-tailed).

A statistically significant correlation was found between soil S levels on crop fields and root HCN levels of bitter cassava varieties cultivated on them (Table 3). The positive correlation coefficient indicates that root HCN levels could increase as S increases in these soils. The relationship with soil S was only observed in bitter cassava varieties, indicating that this association could be variety dependent. Another significant correlation was between root HCN levels of all sampled cassava varieties (bitter and sweet combined) and soil P levels on crop fields. This significant outcome was also positive showing that root HCN levels in all varieties could generally increase as P increases in these soils. None of the significant relationships revealed an increase in cassava root HCN with decreased soil nutrient supply on these soils.

The results of the present study are contrary to other findings on the effects of increased soil S and P on cassava root HCN levels [35,38,42,44]. It is important to note that some of the fields had S and P levels which were high and probably outside the range suitable for optimal cassava growth (Table 2). The high levels of S and P are capable causing nutrient stress and could increase cyanogenic glucosides in cassava.

#### Cyanogenic glucoside contents of sampled cassava roots

Root HCN levels for the varieties sampled from the konzo-affected villages in Mtwara region are given in Table 4.

**Table 4:** Total hydrogen cyanide levels in fresh cassava roots of various cassava varieties cultivated in konzo-affected Mtwara region.

Variety	Type	n	Total HCN levels (mg/kg), fresh weight basis		
			Minimum	Maximum	Mean
*Badi*	Sweet	1	16.8	.	.
*Kigoma*	Sweet	4	43.1	58.1	49.5
*Mnalile Kuchumba*	Sweet	3	25.9	109.8	62.2
*Limbanga*	Bitter	2	114.4	181.9	148.2
*Mohammed Mfaume*	Bitter	1	79.4	.	.
*Musa Saidi*	Bitter	3	51.0	131.1	85.15
*Namanjele*	Bitter	1	44.3	.	.
*Nanjenjeha*	Bitter	3	54.3	144.3	98.1
*Salanga*	Bitter	3	111.3	191.9	147.5

All sampled roots for the bitter cassava varieties, *Limbanga* and *Salanga*, had HCN levels above 100 mg/kg (Table 4). These levels greatly exceeded the current limits of total HCN (less than 50 mg/kg) in fresh cassava roots recommended for their safe consumption [1]. The two varieties thus had a high potential of causing cyanide poisoning if consumed. Some root samples of the bitter varieties *Nanjejeha* and *Namanjele* were however below 50 mg/kg. This showed that some bitter cassava varieties in konzo-affected areas, can also at times contain lower HCN levels and may thus not always be toxic. On the other hand, root samples of the sweet cassava varieties, *Mnalile Kuchumba* and *Kigoma*, had total HCN levels that were above 50 mg/kg. This showed that sweet cassava varieties produced in konzo-affected areas, are also capable of accumulating high levels of cyanogenic glucosides. The observed differences in root cyanogenic glucoside contents between plants of the same variety can probably be explained by differences in nutrient levels of soils on crop fields and also by differences in cassava crop management practices.

### Risk of cassava cyanide intoxication due to soil nutrient levels on crop fields on which cassava is cultivated

Out of the 120 farmers interviewed, 45.8% mentioned that they had at least one household member affected by cassava cyanide intoxication. The intoxication experiences were either acute or had resulted in konzo or even death. Some farmers could remember the exact year in which the cyanide intoxication experience had occurred, this information is shown in Table 5. While a few cases occurred a long time ago, a number of the cases were relatively recent. Only 23.2% of the farmers that had experienced cassava cyanide intoxication in their household were still using the crop field from which the cassava roots that had caused the intoxication had been harvested. It is the soil nutrient levels on these crop fields that were compared with the soil nutrient levels on crop fields of households not affected by cassava cyanide intoxication in the logistic regression analysis.

**Table 5:** Cassava cyanide intoxication experiences as reported by farmers in konzo-affected areas of Mtwara region.

Year	Number of cases
1979	1
1990	1
1999	3
2000	2
2002	1
2003	1
2004	2
2006	1
2007	1
2010	4
2011	2
2012	2
2013	4
2014	3

The results of the logistic regression analysis showing the likelihood of cassava cyanide intoxication occurring due to the soil nutrient levels of crop fields on which cassava was cultivated are shown in Table 6. The logistic regression analysis indicated that pH and Fe were significant predictors (X^2^(*12*) = 26.078, p = 0.010) of cassava cyanide intoxication in konzo-affected Mtwara region. The other 10 soil chemical characteristics (OC, N, P, K, S, Ca, Mg, Cu, Zn and Mn) included in the analysis were not significant predictors of cassava cyanide intoxication. All 12 predictors explained 31.6% (Nagelkerke R^2^) of the variability linking soil nutrient supply to cassava cyanide intoxication. The model had correctly predicted 95.3% of the cases without cyanide intoxication and 34.6% of the cases with cyanide intoxication, and 81.1% of the cases had been correctly predicted.

**Table 6:** Logistic regression analysis for cassava cyanide intoxication in konzo-affected areas of Mtwara region.

			Wald			Odds ratio
Predictor	β	SE	X^2^	df	p-value	Exp(β)
pH	2.640	0.926	**8.124**	1	0.004	14.014
OC	-1.754	2.504	0.491	1	0.483	0.173
N	-0.980	14.205	0.005	1	0.945	0.375
P	-0.071	0.056	1.603	1	0.205	0.931
K	1.867	7.139	0.068	1	0.794	6.468
S	0.090	0.092	0.973	1	0.324	1.095
Ca	-0.061	0.539	0.013	1	0.910	0.941
Mg	-0.446	3.425	0.017	1	0.896	0.640
Fe	0.062	0.026	**5.740**	1	0.017	1.064
Cu	11.745	6.898	2.899	1	0.089	126114.5
Zn	0.017	1.468	0.000	1	0.991	1.017
Mn	0.061	0.035	3.089	1	0.079	1.063
Constant	-16.919	5.594	9.149	1	0.002	0

Note; Cox and Snell R^2^ = 0.209. Nagelkerke R^2^ = 0.316. c-statistic = 81.1%. Hosmer & Lemeshow X^2^(8) = 7.588, p = 0.475. Sensitivity = 34.6%. Specificity = 95.3%. False positive = 30.8%. False negative = 17.4%. Statistical significance was determined at p < 0.05. Al saturation was not included as a predictor variable, as only a few soil samples had a value for it. Values in bold are significant at p < 0.05.

The results of the logistic regression showed that the risk of cassava cyanide intoxication was increased by high levels of pH and Fe in soils on which cassava was cultivated. However, as mentioned previously, cassava cyanide intoxication was only used as a proxy for high (toxic) levels of cyanogenic glucosides in cassava roots in the logistic regression analysis. Thus, replacing the words ‘cyanide intoxication’ with the words ‘high cyanogenic glucoside levels’, the logistic regression analysis can be interpreted as having showed that high soil pH increased the likelihood of getting high cyanogenic glucoside levels in cultivated cassava. High Fe levels in these soils similarly increased the likelihood of getting high cyanogenic glucoside levels in cassava. This implies that cassava cultivated on soils with either high pH or high Fe would be more likely to contain toxic levels of cyanogenic glucosides. These results were different from the findings obtained from the correlation analysis, probably because of the different sensitivities of the two methods.

In agreement with the findings of the present study, cassava root bitterness, which is usually well correlated to root HCN levels, was also reported to increase with increased soil pH on crop fields with pH levels that ranged from 5.8 to 7.8 (soil to water ratio used was 1:2.5) [46]. Although the pH levels on some crop fields were above pH 6.0 in the present study, none of the pH levels exceeded the upper limit of the pH range (4.5–7.0) recommended for optimal cassava growth. With regard to cyanogenic glucoside production, cassava varieties cultivated in Mtwara region may thus have pH requirements below what is recommended as optimal for cassava growth. Near neutral soil pH levels within the optimal range appear to be problematic for these cassava varieties. Increased soil pH was associated with increased levels of K, Ca and Mg in these soils and with decreased OC, Fe and Al saturation (Table 7). An improved supply of K, Ca and Mg should however be beneficial for reducing cyanogenic glucosides in cassava. Reductions in beneficial OC however probably contributed to increasing cyanogenic glucoside levels in cassava roots as pH increased.

**Table 7:** Correlations between soil pH and various soil chemical characteristics on crop fields (n = 112).

Soil characteristic	r	p-value
OC	**-0.257**	0.006
N	-0.077	0.422
P	0.093	0.330
K	**0.343**	0.000
S	-0.020	0.836
Ca	**0.697**	0.000
Mg	**0.548**	0.000
Zn^†^	-0.009	0.928
Cu^†^	-0.060	0.530
Fe^†^	**-0.561**	0.000
Mn^†^	0.121	0.206
Al Saturation^ǂ^	**-0.643**	0.000

Values for correlation coefficients (r) in bold are significant at p < 0.05 using the Pearson correlation (two-tailed).

^ǂ^ For Al saturation, n = 72

^†^ For Zn, Cu, Fe and Mn, n = 111.

In the case of Fe, some crop fields had very high or toxic soil Fe levels (Table 2), making the observed result of increased cyanogenic glucosides with high Fe, somewhat expected. Some levels of Mn were however also very high even if there was no significant association between soil Mn levels and the occurrence of high cyanogenic glucoside levels in cassava. This can be explained by a possible tolerance to high levels of Mn by these cassava varieties, with regard to cyanogenic glucoside production. Back to Fe, the findings of the present study agree with the farmers own perceptions for the causes of increased cassava root bitterness (toxicity) in these areas. The farmers perceived that root bitterness increased in cassava, when it was cultivated on red coloured soils [47]. Red soils contain high levels of oxides or hydroxides of iron and are thus rich in Fe. Furthermore, soils dominated by oxides or hydroxides of Fe are highly weathered and nutrient poor. The observed association between high Fe and increased cassava cyanogenic glucoside levels is hence as a result of the negative effects of low soil fertility.

## Conclusion

Here it was shown that soils in konzo-affected areas of Mtwara region are deficient in multiple soil nutrients and are thus unable to supply cultivated cassava with the nutrients needed to achieve optimal growth. It was also shown that cyanogenic glucoside production in cassava cultivated in konzo-affected areas of Mtwara region is influenced by soil nutrient supply. It is the increased levels of P, S, Fe and of the pH of these soils that is likely to result in high cyanogenic glucoside levels in cassava. Bitter cassava varieties are however more likely to be affected by increased S in soils. As seen from the association between increased cyanogenic glucoside levels and high soil Fe, which is characteristic of nutrient poor soils, low soil fertility could be contributing to increased cyanogenic glucoside levels in cassava cultivated in these areas. Low soil fertility is however not the only possible contributing factor to high cyanogenic glucoside levels in cassava, as above optimal levels of P and S, and near neutral pH levels, are also likely contributors. Although these findings are for cassava cultivated in konzo-affected areas of Mtwara region in Tanzania, similar trends could also be occurring in other konzo-affected areas in sub-Saharan Africa.

## Supporting information

S1 Text Questionnaire. (DOCX)

S2 Text Symbol key for farmer interviews. (DOCX)

S1 Dataset Soil chemical characteristics. (XLSX)

S2 Dataset Cassava root HCN values. (XLSX)

S3 Dataset Farmer interview responses. (XLSX)
