# Supplementary material for: Soil nutrient adequacy for optimal cassava growth, implications on cyanogenic glucoside production: A case of konzo-affected Mtwara region, Tanzania
Source: PLoS One. 2019 May 13;14(5):e0216708. doi: 10.1371/journal.pone.0216708 (PMC6513093; doi:10.1371/journal.pone.0216708)
Supplement: S1 Text — (DOCX) [file pone.0216708.s001.docx]

**QUESTIONNAIRE**

**Families cassava cyanide intoxication experience**

1. Has any member of the family experienced anything strange or any difficulties after consuming cassava? If yes, ask the respondent to describe the symptoms experienced/observed. Check if any of the following is mentioned before indicating **‘Yes’**.

**Symptoms:** Dizziness, headaches, stomach pains, nausea, vomiting, brief confusion (hearing false noises), difficulty in standing, difficulty in speaking (a heavy tongue) or even death.

Yes [ ] No [ ]

1. In which year (or how many years back) was the intoxication experienced? _______________
2. Was the cassava obtained from the families own crop field? Yes [ ] No [ ]

**Location of crop field**

Village: ________________________________ District: _______________­­­­­­___

Southing (S): _______________; Easting (E): __________________; Altitude: ________ m

1. Is this the field from which the cassava roots that caused intoxication had been harvest?

Yes [ ] No [ ]

**Cassava cropping practices**

1. Is/was the cassava planted alone or with other crops? (Observe or ask if the field has been cleared)

1 = mono-cropped [ ] 2 = mixed-cropped [ ] 3 = inter-cropped [ ]

1. If Q1 = 2, list the crops that cassava is/was cropped with?

|  |
| --- |
|  |

1. Is cassava cropped together with cashew? Yes [ ] No [ ]
2. If Q3 = Yes, was the cashew ever dusted with sulphur? Yes [ ] No [ ]
3. At which months was cassava weeded? (Ask for cassava that was planted this cropping season) (Tick months)

| **TIME PERIOD** | **Wet season** | | | | | **Dry season** | | | | | | | **Wet season** | | | | | **Dry season** | | | | |
| --- | --- | --- | --- | --- | --- | --- | --- | --- | --- | --- | --- | --- | --- | --- | --- | --- | --- | --- | --- | --- | --- | --- |
|  | **2013** | | | | | **2013** | | | | | | | **2014** | | | | | **2014** | | | | |
|  | **Dec** | **Jan** | **Feb** | **Mar** | **Apr** | **May** | **Jun** | **Jul** | **Aug** | **Sep** | **Oct** | **Nov** | **Dec** | **Jan** | **Feb** | **Mar** | **Apr** | **May** | **Jun** | **Jul** | **Aug** | **Sep** |
| **Tick month** |  |  |  |  |  |  |  |  |  |  |  |  |  |  |  |  |  |  |  |  |  |  |

**Soil nutrient management**

| Questions | Response | Write comments made on fertiliser use and if used mention type used, when used, and on which crop |
| --- | --- | --- |
| 1. Do you use chemical fertilisers on any crops grown? | Yes [ ]  No [ ] |  |
| 1. Do you apply manure on any crops grown? | Yes [ ]  No [ ] |  |
| 1. Do you grow legumes only to later cut them before they flower and incorporate them into the soil or leave them over the soil (*green manure*) on any crop grown? | Yes [ ]  No [ ] |  |
| 1. Do you apply compost? | Yes [ ]  No [ ] |  |

1. Do you practice the slash and burn practice? Yes [ ] No [ ]
2. If Q10 = Yes. How many years do you normally crop land before you leave it to fallow (rest)?

________ years

1. If Q10 = Yes. How long do you leave the land fallow (to rest)? ___________ years
