# Supplementary material for: Soil nutrient adequacy for optimal cassava growth, implications on cyanogenic glucoside production: A case of konzo-affected Mtwara region, Tanzania
Source: PLoS One. 2019 May 13;14(5):e0216708. doi: 10.1371/journal.pone.0216708 (PMC6513093; doi:10.1371/journal.pone.0216708)
Supplement: S2 Text — (DOCX) [file pone.0216708.s002.docx]

**SYMBOL KEY FOR FARMER INTERVIEWS**

**Some symbols**

888 = Not applicable; 999 = Blank/missing; 777 = I do not know; 1 = Yes; 0 = No

Q1 = Intoxication

Q2 = year of intoxication experience

Q3 = cassava picked from household field

Q4 = field from which causative roots were picked or ActualField

Q10 = inorganic fertiliser use

Q11 = animal manure use

Q5 = cropping method

Q6

| Q6maiz | Maize |
| --- | --- |
| Q6sorg | Sorghum |
| Q6mille | Millet |
| Q6rice | Rice |
| Q6pigp | Pigeon peas |
| Q6cow | Cow peas |
| Q6bamb | Bambara nuts |
| Q6gnut | Groundnuts |
| Q6ses | Sesame |
| Q6swepot | Sweet potatoes |
| Q6pum | Pumpkin |
| Q6yam | Yams |
| Q6pineap | Pineapple |
| Q6cash | Cashew |
| Q6bana | Banana |
| Q6mango | Mango |

Q7 = is cassava intercropped with cashew

Q8 = cashew intercropped with cassava sulphur dusted

Q9 = number of weeding in first three months

Q14 = use of slash and burn practice

Q15 = Cropping cycle in years

Q16 = Fallow length in years
